# Evaluation of Protein Ion Relative Ratio Quantification in Top-down Electrospray Ionization-Mass Spectrometry Using Site-Specific Acetylated Recombinant Histone H3 Proteoforms

**DOI:** 10.1021/jasms.5c00079

**Published:** 2025-09-05

**Authors:** Kin-Wing Lui, Sai-Ming Ngai, Ting-Fung Chan

**Affiliations:** † School of Life Sciences, 26451 The Chinese University of Hong Kong, Sha Tin, N.T., Hong Kong, 999077; ‡ State Key Laboratory of Agrobiotechnology, and School of Life Sciences, The Chinese University of Hong Kong, Shatin N.T., Hong Kong, 999077

**Keywords:** Top-down proteomics, quantitative proteomics, proteoforms, histone H3, protein ion relative ratio

## Abstract

Electrospray ionization (ESI)-mass spectrometry (MS) is a key platform for analyzing post-translationally modified proteins. With continuous advances in MS instruments and data analysis methods, top-down analysis of intact proteoforms has become highly feasible. To accurately quantify proteoforms with varying post-translational modifications (PTMs), the influence of PTMs on the ESI-MS detection efficiency must be considered. Two decades ago, Kelleher and co-workers proposed using protein ion relative ratios (PIRRs) and fragment ion relative ratios (FIRRs) in ESI-MS for proteoform quantification. While FIRR quantification has been extensively studied, the reliability of PIRR quantification—particularly for proteoforms with varying PTM degrees—remains under-evaluated. In this study, we further validated the fidelity of PIRR quantification in top-down ESI-MS using various site-specifically acetylated recombinant histone H3 proteoforms. These proteoforms, carrying varied degrees of acetylation, were produced using an orthogonal translation system that incorporates acetyllysine at the amber stop codons. After absolute quantification by UV spectrophotometry, samples were mixed in isometric ratios and analyzed by either direct infusion-ESI-MS or weak cation exchange/hydrophilic interaction-ESI-MS. Our results show that PIRRs match theoretical ratios regardless of the acetylation degree or site. These findings reinforce the validity of top-down proteoform quantification, especially for histone proteins.

## Graphical abstract

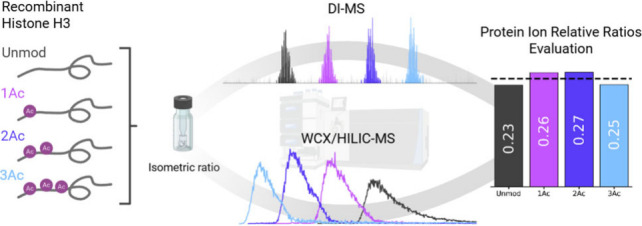

## Introduction

Electrospray ionization (ESI)-mass spectrometry (MS) is a powerful analytical approach for identifying and characterizing peptides and proteins.^1^ ESI efficiently converts liquid chromatography (LC) eluates into gas-phase ions for MS analysis. When coupled with nanoflow LC and nano-ESI, this approach enables sensitive, high-throughput separation and detection of complex biological samples.^2^ LC-ESI-MS (LC-MS), combined with optimized sample preparation, front-end separation techniques, and advanced bioinformatics, has become a cornerstone of quantitative proteomics. Traditionally, quantitative proteomics relied on stable isotopic labeling^3^ or fragmentation-cleavable isobaric tags,^4–6^ which enabled proportional quantification across samples by comparing ion signal between labeled molecules. However, improvements in MS speed, accuracy, and resolution have made label-free quantification (LFQ) a viable alternative. In LFQ, samples are analyzed individually, and their ion signals are integrated, normalized, and compared across data sets.

In a typical bottom-up proteomics workflow, proteins are first digested into peptides (typically via trypsin) and then analyzed by LC-MS. To quantify proteins, peptide signals must be filtered and aggregated into protein abundances. However, peptide detection efficiencies can vary significantly due to differences in ionization efficiencies, necessitating sophisticated normalization to minimize quantification bias.^7–11^ Recent advances—including data-independent acquisition (DIA), ultrahigh-speed MS instrumentation, and improved computational tools—have significantly enhanced the efficiency and robustness of LFQ proteomics.^12–14^

In contrast to bottom-up proteomics, the top-down approach analyzes proteins in their intact and undigested form.^15^ This method preserves the full chemical modifications landscape and sequence variation along the protein backbone, enabling investigation at the proteoforms level, which are the distinct molecular species derived from a single gene.^16^ Quantitative analysis, including LFQ, has also been implemented in top-down proteomics.^17–19^ This approach offers unique advantage—by measuring intact proteoform directly, it eliminates quantification biases inherent to peptide-based methods such as incomplete digestion, shared peptides, or missing peptides between runs.^20^

In quantitative proteomics, protein abundance comparisons across samples are fundamental. However, as noted earlier, ESI-MS detection efficiencies vary between analytes, making abundance comparisons across peptides and proteins unreliable without proper signal correction and normalization. This challenge is well-characterized in peptide-based studies, where post-translationally modified peptides exhibit significantly variable signal responses depending on modification type, position, or degree.^21–23^ The observed signal variation primarily stems from the differences in analyte ionization efficiency, which correlate strongly with the physicochemical properties. The equilibrium partitioning model of ESI^24–26^ explains that less polar analytes partition preferentially to the droplet surface, acquiring excess charges and ionize more efficiently. Similarly, more basic analytes demonstrate enhanced ionization in positive-mode ESI due to their greater proton affinity.^25,27–31^ While these principles provide a framework for understanding ESI behavior, the precise ionization mechanisms remain incompletely characterized, particularly for peptides, unfolded proteins, and native proteins.^30,32,33^

A pivotal 2006 study by Kelleher and colleagues demonstrated that unfolded intact proteoforms exhibited remarkably similar detection efficiences in ESI-MS.^23^ Through histidine-to-alaine mutations mimicking lysine acetylation’s charge-neutralizing effects, they showed that the precursor ion intensity ratio between pseudoacetylated and unmodified histone H4 closely matched their actual mixture ratios. This finding suggested that unlike peptides, intact proteins—with their greater size and abundance of basic residues—maintain consistent ionization efficiency despite sequence variations or post-translational modifications (PTMs).^22,34^ The study further confirmed that fragment ion intensity ratios accurately represented coisolated precursor ions, establishing two key quantitative metrics: protein ion relative ratios (PIRRs) and fragment ion relative ratios (FIRRs). Over the past two decades, these concepts have been extensively refined and applied, forming the foundation of modern quantitative top-down proteomics.^35–41^ This approach is particularly valuable for histone epigenetics research, where it enables precise measurement of PTM stoichiometry and dynamics^34,42^ —a capacity crucial for understanding complex PTM “cross-talks”,^43^ especially on histone H3.^44^

While PIRR and FIRR quantifications are interdependent in top-down analysis, their underlying principles differ fundamentally. PIRR quantification depends on the assumption that proteoforms exhibit comparable ESI-MS detection efficiencies, whereas FIRR quantification relies on the consistency of the fragmentation efficiency and the robustness of multiplexed MS/MS spectral analysis. While the challenges associated with FIRR quantification have been thoroughly addressed,^23,36–38,40,41,45–47^ the fidelity of PIRR quantification—particularly for proteoforms with varying modification degrees—remains understudied to our knowledge.

Building upon the foundational work with the singly pseudoacetylated proteoform conducted two decades ago, we systematically evaluated PIRR quantification accuracy for intact histone H3 proteoforms containing genuine, site-specific acetylations at varied degrees. Using an orthogonal translation system incorporating acetyllysine via amber stop codon suppression,^48^ we produced full-length H3 proteoforms with defined single or multiple acetylation sites. Absolute quantification by UV spectrophotometry enabled precise mixing of proteoforms in predetermined ratios (1:1:1:1 or 1:1) for analysis by two complementary approaches—conventional direct infusion-ESI-MS (DI-MS) and online weak cation exchange/hydrophilic interaction LC-ESI-MS (WCX/HILIC-MS).^49–51^ Our results demonstrated that all measured PIRRs closely matched their theoretical ratios, with proteoform detection efficiencies remaining consistent regardless of the acetylation degree or location. These findings provide robust experimental validation for PIRR quantification in top-down ESI-MS analysis, particularly for studying post-translationally modified histone.

## Experimental Section

### Histone H3.3 C120A M120A (H33CM) Expression Plasmid

This study used *Homo sapiens* histone H3.3 (H3–3A) as the model protein due to its minimal cysteine and methionine content among canonical histone H3 variants (H3.1, H3.2, and H3.3). The remaining C110 and M120 residues were substituted with alanine (yielding H33CM) to prevent oxidation-related spectra complications.^51,52^ A His6-TEV tag was fused to the N-terminus (Sequence S1) for purification and tag cleavage, producing two forms: His6-TEV-H33CM (full construct) and H33CM (TEV-cleaved protein retaining one N-terminal glycine). The His6-TEV-H33CM gene was synthesized by GenScript and cloned into pET-11a under T7/Lac promotor control (Figure S1). Site-directed mutagenesis (QuikChange Lightning Kit, Agilent) introduced amber codons at lysine 9, 79, 115, 9&36, or 9&27&36 (primers in Table S1), generating the following proteoforms: unmodified H33CM (“unmod”), H33CMK9ac (“1Ac”), H33CMK9acK36ac (“2Ac”), H33CMK9acK27acK36ac (“3Ac”), H33CMK79ac (“K79ac”), and H33CMK115ac (“K115ac”). All positions follow canonical histone H3 numbering despite the +1 offset from retained TEV glycine, ensuring consistency with established nomenclature.

### Orthogonal Translation Plasmids

The orthogonal translation plasmid pTECH-chAcK3RS­(IPYE)^48^ for acetyllysine incorporation via amber suppression was a gift from David Liu (Addgene plasmid #104069; http://n2t.net/addgene:104069; RRID:Addgene_104069).

### Protein Expression

For the unmodified proteoform expression, BL21­(DE3) competent cells were transformed with the pET-11a-His6-TEV-H33CM plasmid via a CaCl_2_ heat shock. Transformant were cultured in LB medium supplemented with 100 μg/mL ampicillin (Amp) at 37 °C. Protein expression was induced using 0.2 mM isopropyl β-d-1-thiogalactopyranoside (IPTG) when the optical density at 600 nm (OD_600_) was >0.6.

For acetyllysine incorporation via amber suppression, B-95.ΔA cells^53^ were cotransformed with both the pTECH-Ack3RS­(IPYE) and amber-mutated pET-11a-His6-TEV-H33CM plasmids. The B-95.ΔA strain was provided by the RIKEN BRC through the National BioResource Project of MEXT/AMED Japan. Transformed cells were cultured in LB medium containing 100 μg/mL Amp and 25 μg/mL chloramphenicol (*Cm*), with the *Cm* concentration reduced to 3 μg/mL during expression. When cultures reached OD_600_ > 0.6, they were supplemented with 20 mM nicotinamide (Sigma-Aldrich) and *N*_ε_-Acetyl-l-Lysine (AcK; Sigma-Aldrich) at concentrations detailed in the Results and Discussion section. Protein expression was induced with IPTG 30 min later.

Complete protocols for bacterial transformation and recombinant protein expression are provided in the Supplementary Methods.

### Protein Purification

Cell pellets were resuspended in 6 M urea Tris-HCl buffer (pH 8) and lysed by sonication. After centrifugation, the clarified lysate was purified using a Ni-NTA Spin Column (Qiagen). The urea concentration in the eluate was reduced to 1 M by using a 10 kDa MWCO centrifugal concentrator (Amicon Ultra-0.5 Centrifugal Filter Unit Ultracel-10, Milipore) prior to TEV protease cleavage. Cleaved proteins were further purified by reverse-phase HPLC (Figure S2), with final products analyzed by DI-MS with electron transfer dissociation (ETD)-MS/MS. Proteoform spectrum matching was performed using Informed-Proteomics^54^ (version 1.0.7017), with respresentative proteoform-spectrum matches shown in Figure S3. Sample purity was assessed via TopFD (version 1.7.8, part of the TopPIC suite^55^), with precursor features summarized in Table S2.

Detailed purification and analysis protocols are provided in the Supplementary Methods.

### UV Spectrophotometry

Histone H3 concentrations were quantified by A_210_ absorbance using a NanoDrop One^C^ Microvolume UV–vis Spectrophotometer (Thermo Fisher Scientific Inc.), with 2 μL samples measured in 0.1% formic acid (FA). A calibration curve was generated using serial dilutations of commercial recombinant human histone H3.1 protein (recombinant Histone H3 (1–136) human, expressed in *Escherichia coli*, ≥95% SDS-PAGE, 100 μg, Sigma-Aldrich) (Figure S4A–B). HPLC-purified samples were vacuum-dried, reconstituted in 100 μL 0.1% FA, and quantified before being diluted to 0.1 μg/μL (Figure S4C). Three isometric test mixtures were prepared: unmod/1Ac/2Ac/3Ac, unmod/K79ac, and unmod/K115ac.

### DI-MS

DI-MS was performed using a modified UltiMate 3000 RSLCnano system with ProFlow technology (Thermo Fisher Scientific Inc.) coupled to an Orbitrap Fusion Lumos Tribrid MS instrument (Thermo Fisher Scientific Inc.) via a Nanospray Flex ESI source (Thermo Fisher Scientific Inc.). The system incorporated a C4 trap cartridge (PepMap 300, 5 mm × 0.3 mm I.D., particle size 5 μm, pore size 300 Å, Thermo Fisher Scientific Inc.) connected to a switching valve with two operational modes: in *load* position, the cartridge linked to the loading pump and waste outlet for sample loading; In *elute* position, it connected to the nanoflow pump and MS inlet for analysis.

For DI-MS analysis, 200 ng of the sample was loaded onto the trap cartridge at 5 μL/min using 0.1% FA for 5 min, followed by isocractic elution into the ESI-MS system at 0.4 μL/min with 70% ACN/0.1% FA for 20 min after valve switching.

ESI-MS was operated in positive ion mode with a 2.0 kV spray voltage, 300 °C ion transfer tube temperature, and low pressure mode. Infusion mode was disabled, and advanced peak determination was enabled. The default charge state was set to 10.

The MS scan properties were as follows: 30% RF lens, no source fragmentation, Orbitrap resolution of 120,000 (fwhm at 200 *m*/*z*), scan range of 500–2000 *m*/*z*, normalized automatic gain control target of 125% (equivalent to 5e5), maximum injection time of 50 ms, three microscans, and profile mode data collection.

DI-MS analyses of the proteoform mixtures were performed in triplicate.

### WCX/HILIC–MS

WCX/HILIC-MS was performed using a setup similar to DI-MS. The trap cartridge was replaced with a PolyCAT-A trap column (50 mm × 0.15 mm I.D.), followed by a PolyCAT-A analytical column (150 mm × 0.075 mm I.D.) connected via the switching valve. The analytical column effluent was directed through the MS inlet capillary to the ion source. Both columns (PolyLC Inc.) had a particle size of 3 μm and a pore size of 1500 Å. The mobile phases consisted of Buffer A (70% ACN/1% FA) and Buffer B (60% ACN/10%FA).^49–51^

For WCX/HILIC-MS analysis, 200 ng of sample was loaded into the trap column at 5 μL/min (buffer A) for 5 min using the loading pump. The trapped proteoforms were then eluted through the analytical column to the ESI-MS system using the nanoflow pump with the following gradient: 0% to 100% B in 100 min, followed by an isocratic hold at 100% B for 30 min. The nanoflow pump maintained a constant flow rate of 0.4 μL/min throughout the separation.

All WCX/HILIC-MS analysis was performed in triplicate using the same MS parameters as for DI-MS, with the addition of MS/MS acquisition. Detailed parameters are provided in Supplementary Methods.

Milli-Q water and HPLC-grade ACN (or higher purity) were used as solvents for all experiments.

### Data Analysis

PIRRs were calculated through the following workflow: sample. raw files were centroided and converted to. mzML format using MSConvert (version 3.0.24117–481ff6c; part of the ProteoWizard Suite^56^). Deconvolution was performed with TopFD using these parameters: peak error tolerance of 0.02 *m*/*z*, MS1 and MS/MS signal-to-noise ratio of 3, precursor window of 0.4 *m*/*z*, maximum charge of 30, minimum scan number of 3, disabled MS-Deconv score, and ECScore cutoff of 0.5, and enabled final filtering. Subsequent data analysis was conducted using Python scripts.

The abundance of proteoform *i* (*Ab*(*Pr*_*i*_)) was quantified as the area under curve (AUC) of its precursor ions calculated via the composite trapezoidal rule (numpy.trapz):

$$Ab\left({\mathrm{Pr}}_{i}\right)=\underset{k}{\sum }\frac{1}{2}(PInt\left({\mathrm{Pr}}_{i},t_{k-1}\right)+PInt\left({\mathrm{Pr}}_{i},t_{k}\right))\left(t_{k}-t_{k-1}\right)$$

where *PInt*(*Pr*_*i*_ *,t*_*k*_) denotes the precursor ion intensity of proteoform *i* at elution time *t*_*k*_. All *k* MS scans between the minimum and maximum elution times were considered. For DI-MS, the elution time range was set to 5–25 min; for WCX/HILIC-MS, it was set to 25–75 min.

For each MS scan, *PInt*(*Pr*_*i*_ *,t*_*k*_) was obtained by summing intensities of all precursor ions within ± 1.5 Da of the proteoform’s monoisotopic mass (deconvoluted by TopFD). All charge states were considered unless specified otherwise:

In the MS scan at time *t*_*k*_,

$$PInt\left({\mathrm{Pr}}_{i},t_{k}\right)=\underset{mass}{\sum }\underset{charge}{\sum }Intensity\left({Ion}_{mass,charge}\right)$$

for *mass* ± 1.5 Da from the monoisotopic mass of *Pr*_*i*_, and *charge* within the specified charge state range.

The PIRR of proteoform *i* was then calculated as the ratio of its abundance to the total abundance of all *n* proteoforms in the sample:

$${PIRR}_{i}=\frac{Ab\left({\mathrm{Pr}}_{i}\right)}{\sum Ab({\mathrm{Pr}}_{n})}$$

The calculated proteoform abundances and PIRRs are provided in Tables S3 and S4.

MS spectra illustrations were generated from representative scan data, while MS/MS illustrations were created by using averaged MS/MS spectra. Raw data export (for both MS and MS/MS) and MS/MS spectral averaging were performed by using FreeStyle software (ThermoFisher Scientific). Final ion annotation and figure generation were performed by using Python scripts.

All spectral data and analysis results were uploaded to ProteomeXchange Consortium via the PRIDE^57^ partner repository with the data set identifier PXD064777.

## Results and Discussion

### Acetylated Histone H3 as the PIRR Quantification Evaluation Model

Histone H3 serves as an ideal model system for this investigation due to its prominent role in top-down proteomics applications, particularly for characterizing combinational PTMs. As the histone variant with the most diverse repertoire of biologically significant PTMs, H3 provides an optimal platform for evaluating the PIRR quantification. This evaluation benefits both fundamental proteomics methodology and histone epigenetics research.

Using acetylated histone H3 proteoforms as models, the study specifically evaluates the effect of PTM-induced changes comparable to lysine acetylation—a modification that neutralizes a positive charge while increasing hydrophobicity.^58,59^ We generated proteoforms with up to three acetylations to assess how cumulative modifications influence the detection efficiency. To test for sequence-position effects, we introduced acetylations in three distinct domains: the N-terminal tail, the core region, and the C-terminal tail.

Furthermore, this study focuses on proteins exhibiting high detection efficiency under denaturing acidic ESI-MS conditions. Current ESI-MS theory suggests that proteins with length and basic residue content similar to or exceeding those of histone H3 should demonstrate comparable detection efficiency.^29,30,34^ While all canonical histones likely meet these criteria, we emphasize the importance of empirical validation with ground truth samples before performing PIRR quantification to either nonhistone proteins or proteoforms containing bulkier modifications such as ubiquitination, where additional mass and structural complexity may alter ionization behavior.

### Recombinant Histone H3 Proteoforms with Site-Specific Modifications

Besides evaluating ESI-MS quantification, this study also developed a protocol for producing pure, well-defined histone proteoforms. As recently highligted by Searfoss et al.,^60^ recombinant histone proteoforms serve as invaluable controls for methodological developments in both epigenetics studies (e.g., investigations of protein binding specificity and enzymatic activities) and LC-MS applications (e.g., retention time charaterization, fragmentation pattern analysis, PTM localization, and quantificative method validation).

Currently, commercially available modified full-length histones exhibit limited PTM variability. These products are mainly generated using either expressed protein ligation (require peptide synthesis)^61^ or methylated lysine analog technologies (which substitutes methylated aminoethyl-cysteine for genuine methyllsine).^62,63^ To overcome these limitations, we implemented an orthogonal translation system (OTS) capable of incorporating authentic site-specific modifications with minimal specialized equipment. This approach utilizes engineered tRNA/tRNA-transferase pairs (sometimes including modified elongation factors) to precisely incorporate noncanonical amino acids at targeted codons during translation.^64^ Several OTS plasmids encoding distinct tRNA/tRNA transferase pairs are available through Addgene. For acetylated histone H3 production, we employed David Liu’s pTECH-chAcK3RS­(IPYE) OTS plasmid^48^ (Addgene #104069). It contains a tRNA_CUA_ recognizing amber (UAG) stop codon, and an optimized *N* ^ε^-acetyllysyl-tRNA synthetase (AcKRS) specifically charging the tRNA with *N* ^ε^-acetyllysine (AcK). The histone H3 gene was site-specifically modified to incorporate amber codons using the QuikChange Lightning Site-Mutagenesis kit. To maximize the amber suppression efficiency and protein yield, we utilized the B95.ΔA expression host (a BL21­(DE3) derivative developed by RIKEN). The engineered strain features knockout of release-factor 1 (*RF-1*) to prevent premature translation termination, and replacement of genomic amber codons in essential genes with alternative stop codons (TAA or TGA).^53^

Existing protocols typically use 1 mM AcK for orthogonal translation and 25 μg/mL *Cm* for cell culture maintenance.^48,65^ However, our optimization revealed that detectable target protein expression (assessed by SDS-PAGE; Figure S5A) required at least 5 mM AcK. Furthermore, reducing *Cm* concentration from 25 μg/mL to 3 μg/mL in the expression culture significantly enhanced yields (Figure S5B), likely due to alleviation of *Cm*’s ribosome-blocking effects.^66^ Under optimized conditions (3 μg/mL *Cm*, 20 mM AcK, and 20 mM nicotinamide), we achieved comparable yields (∼6 μg/mL culture, quantified after HPLC) between singly acetylated and unmodified histone H3.

While 20 mM AcK enabled expression of singly acetylated H3, this concentration failed to produce detectable yields for multiacetylated proteoforms. After testing various conditions, we found that histone H3 with two and three amber mutations required 100 mM AcK and 200 mM AcK, respectively, to achieve expression yield comparable to unmodified H3 (Figure S5C, D)

The expressed recombinant proteoforms were individually analyzed by DI-MS, each yielding sharp precursor ion signals (Figure 1 “MS”, Figure S6). TopFD analysis indicated that the target proteoforms represented 70–80% of total ion intensity. Two prominent off-target species were observed with mass shifts of approximately +43 and +98 Da relative to the targeted masses. The +43 Da shift suggests possible disodium adduct formation (+2Na),^67^ while the +98 Da shift may indicate phosphoric acid adduction (+H_3_PO_4_).^68^ These off-target species collectively accounted for 9–17% of total ion intensities across samples (Table S2, Figure S6).

**Figure 1:**
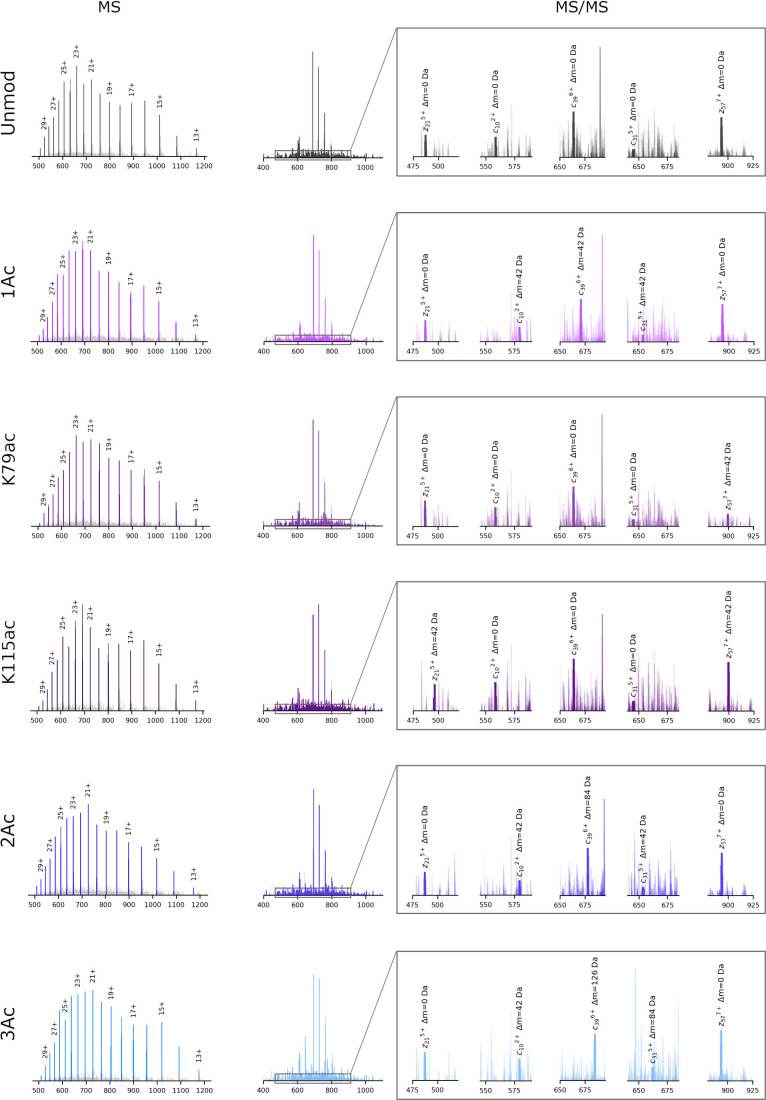
MS and MS/MS characterizations of the recombinant histone H3 proteoforms. From top to bottom, unmodified H33CM (Unmod), H33CMK9ac (1Ac), H33CMK79ac (K79ac), H33CMK115ac (K115ac), H33CMK9acK36ac (2Ac) and H33CMK9acK27acK36ac (3Ac) proteoforms. Target proteoform ions are shown in unique colors. Unrelated signals are shown in pale gray. Left panel (“MS”), representative MS spectra (expanded views in Figure S6). Right panel (“MS/MS”), averaged ETD spectra with five representative fragment ions (*c*_10_^2+^, *c*_31_^5+^, *c*_39_^6+^, *z*_57_^7+^, and *z*_21_^5+^) highlighted in zoomed views (ordered by *m*/*z*). Selected for their consistent detection across all proteoforms, these fragments are plotted as thick bars to enable a direct comparison of acetylation patterns. Other matched fragments are displayed at a reduced opacity. All proteoforms retain an N-terminal glycine from TEV cleavage, shifting residue numbering (and their corresponding ion indices) by +1 compared to canonical histone H3. For clarity, the canonical histone nomenclature is maintained throughout the study. Mass tolerances: 1.5 Da (precursors) and 20 ppm (fragments).

ETD-MS/MS analysis generated specific fragment patterns that unambiguously identified acetylation sites (Figure 1 “MS/MS”) while achieving near-complete sequence coverage (Figures S3 and S7). While fragment patterns showed general consistency across proteoforms, we observed intensty variations for specific ions. These differences should be interpreted with caution because the ETD parameters were not optimized for quantitative analysis, and accurate FIRR quantification requires coisolation and cofragmentation of the proteoforms. Therefore, these ETD data are primarily useful for proteoform characterization. Detailed investigation of ETD-based FIRR quantification can be found in other studies.^41,46^

### Evaluating PIRR Quantification for Histone H3 Proteoforms with Varied Acetylation Degrees

Proteoform mixtures with defined ratios were prepared by first quantifying the recombinant proteoforms via UV spectrophotometry (A_210_ nm, peptide bond absorbance^69^) using a calibration curve generated from commercial human histone H3.1 standards. Given the 94% sequence identity between our recombinant histone H3 and human H3.1 (Sequence S1), we assumed comparable A_210_ nm absorption properties. After adjusting all recombinant proteoforms to equal concentrations (Figure S4), we prepared three isometric mixture series for PIRR quantification: unmod/1Ac/2Ac/3Ac to test the effects of increasing acetylation degree, unmod/K79ac to examine core region acetylation, and unmod/K115ac to assess C-terminal tail acetylation.

The samples were analyzed using two ESI-MS platforms: DI-MS and WCX/HILIC-MS. For DI-MS, we implemented a modified setup using a C4 cartridge to retain samples injected from the autosampler, followed by simultaneous elution to the ion source with 70% acetonitrile. This approach reduces sample consumption compared to traditional DI-MS while enabling online desalting. Although DI-MS represents the simplest approach, WCX/HILIC offers superior separation of histone proteoforms through a combined positive charge retention and hydrophilic interaction. Early WCX/HILIC was incompatible with ESI due to nonvolatile salts, limiting their use to off-line fractionation prior to MS analysis.^35,70,71^ Critical developments by Garcia’s group (middle-down analysis^72^) and Paša-Tolić’s group (top-down analysis^49^) developed ESI-compatible WCX/HILIC methods. Garcia’s method employs dual pH and polarity gradients to separate analytes by charge and hydrophilicity, while Paša-Tolić’s approach utilizes high concentrations of acetonitrile to enhance hydrophilic retention, coupled with a volatile formic acid gradient for charge-based elution. Our 2021 comparison of these methods with reversed-phase LC for histone H3 proteoforms demonstrated that Paša-Tolić’s “organic rich” WCX/HILIC-MS provided superior proteoform separation and characterization.^51^

For both DI-MS and WCX/HILIC-MS analyses, proteoform abundances were quantified by integrating signal intensities across the elution profile. PIRRs were derived by normalizing individual proteoform abundances against the sum of all targeted proteoforms in the mixture. Complete data sets of all proteoform abundances and calculated PIRRs are provided in Tables S3 and S4.

All proteoforms coeluted with homogeneous profiles in DI-MS (Figure 2, second row), and PIRRs closely matched theoretical isometric ratios (Figure 2, first and third rows). However, a minor bias toward higher acetylation states was observed, quantified both as absolute errors (*Observed PIRR – Theoretical PIRR*) and percent errors $\left(\frac{\mathrm{Observed PIRR}-\mathrm{Theoretical PIRR}}{\mathrm{Theoretical PIRR}}\times 100\%\right)$ . For the unmod/1Ac/2Ac/3Ac sample, the 1Ac proteoforms showed an absolute error of 0.0044 (1.76% error), while 2Ac and 3Ac proteoforms exhibited larger deviations of 0.0162 (6.48%) and 0.0175 (6.98%), respectively. Similar trends appeared in unmod/K79ac (0.012, 2.32%) and unmod/K115ac (0.05, 10.09%) sets (Table S3). Interference from the +2Na adducts (+43.96 Da) likely explains this systematic bias, as their small mass difference from acetylation (+42.01 Da) could artificially inflate signals for acetylated species (Figure S6). The effect showed strong dependence on sample composition: in mixtures where the unmodified proteoform dominated (Figure S8 “75%” and “95%”, Table S4), only the 1Ac proteoform exhibited elevated PIRRs—consistent with interference from +2Na adducts of the predominant unmodified precursors. In the 5% unmodified proteoform mixture (Figure S8 “5%”, Table S4), PIRR inflation occurred exclusively in 2Ac and 3Ac proteoforms. At this low proportion, interference from unmodified proteoform +2Na adducts became negligible, making the +2Na adducts of 1Ac and 2Ac proteoforms the sole observable source of signal inflation.

**Figure 2:**
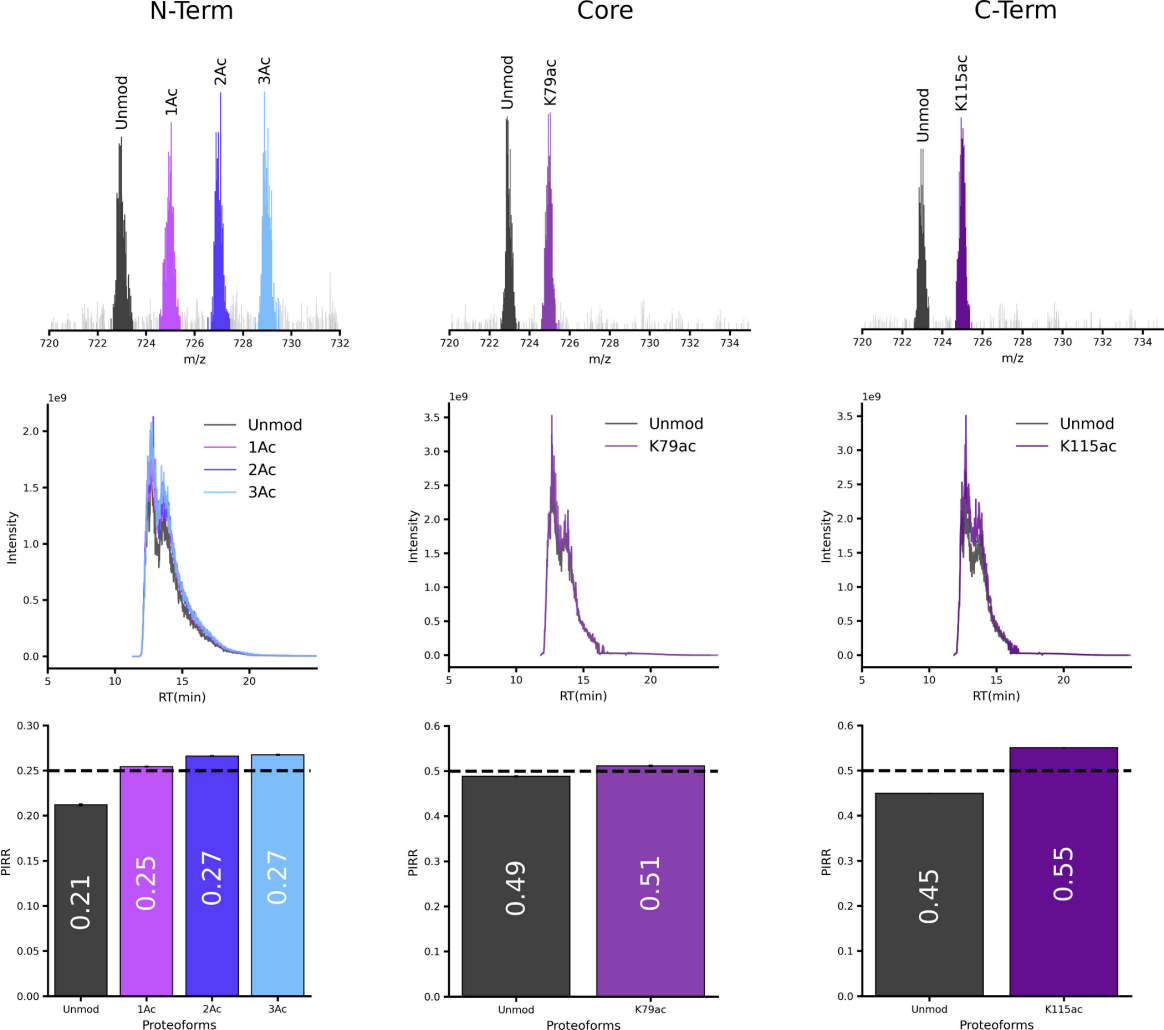
DI-MS analysis of isometrically mixed histone H3 proteoforms with varied acetylation degrees. Color scheme matches Figure 1. (Top) Representative precursor ion signals (21+ charge state shown. All charge states included in calculations). (Middle) DI-MS chromatograms. RT: retention time in minutes. (Bottom) Observed PIRRs (mean ± SD, *n* = 3) with theoretical ratios indicated by dashed lines. Numerical PIRR values are labeled within each bar. Error bars appear minimal due to minor experimental deviations.

WCX/HILIC separated proteoforms by the N-terminal acetylation state (Figure 3, top row). Retention behavior was position-dependent, with acetylation sites distal to the N-terminus having progressively weaker effect on separation. These observations align with the established WCX/HILIC mechanism on histones: the stationary phase primarily interacts with the basic, hydrophilic N-terminal tail, making modifications on this region more influential on retention time.^73^

**Figure 3:**
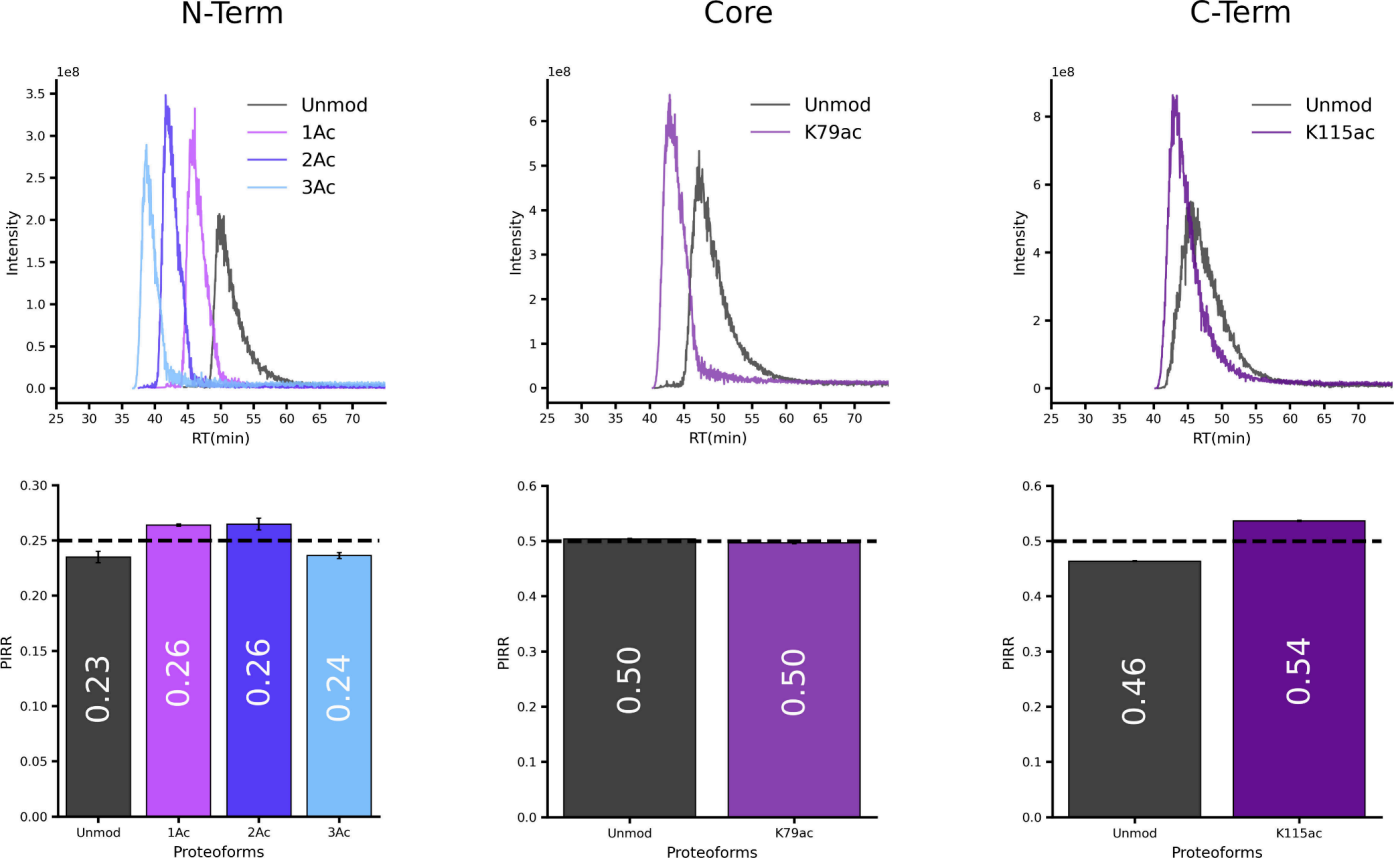
WCX/HILIC-MS analysis of isometrically mixed histone H3 proteoforms with varied acetylation degrees. The display format is as follows: Figure 2, excluding the representative precursor ion illustrations.

WCX/HILIC-MS demonstrated PIRR quantification performance comparable to DI-MS, with observed ratios closely matching theoretical values (Figure 3, bottom row; Table S3). Contrast to our initial hypothesis that LC separation would mitigate ion adduct interference, we still detected PIRR inflation for acetylated proteoforms (Figure 3, Figure S9, Table S4). In mixtures dominated by the unmodified proteoform (Figure S9 75% and 95%, Table S4), the 1Ac proteoform consistently showed elevated signals. This suggests either coelution of +2Na adducts with acetylated proteoforms or bacterial host-derived spontaneous acetylation. ETD-MS/MS ruled out cross-sample contamination, as the inflated 1Ac signals lacked detectable acetylation at expected sites. The data cannot confirm whether the inflation stems from +2Na adduct interference or spontaneous modification; both remain plausible.

In addition to the interference effects, WCX/HILIC-MS showed minor under-representation of the 3Ac proteoforms. This bias likely originates from differential binding during sample loading as higher acetylation degrees reduce WCX/HILIC retention.

Despite these interferences, all observed PIRRs closely matched their theoretical values, with accuracy comparable to the singly pseudoacetylated histone H4 reported in Pesavento et al., 2006, (−0.01 absolute error and – 1.7% percent error for observed vs theoretical PIRRs of 0.58 vs 0.59). Notably, when contrasted with the significant quantification errors documented for bottom-up^23,74^ and middle-down analyses^75^ of histone peptides, our results demonstrate superior reliabilty and accuracy of top-down PIRR quantification for intact histone proteoforms.

The precision of PIRR quantification in our data set is particularly notable. Prior to PIRR normalization, proteoform abundances displayed variable reproducibility across replicates with coefficients of variation (CV) ranging from 0.2% to 14%. Following PIRR normalization, CV values improved substantially, with most measurements below 1% and a maximum observed CV of only 2.2% (Table S3). This marked improvement underscores how abundance normalization enables prescise interanalysis comparisons in MS experiments.

In this study, proteoform abundances were calculated by integrating signals across all of the precursor charge states. For comparison, we also performed calculations using either individual charge states or the three most abundant charge states. The results remained consistent across the methods, with significant deviations only occurring when calculations were restricted to the least abundant charge states (e.g., charge 13+ or 30+ in DI-MS; Figures S10 and S11).

## Conclusions

Top-down ESI-MS quantification using PIRR and FIRR was proposed over two decades ago. While FIRR quantification has seen significant methodological advances, systematic evaluation of PIRR quantification fidelity—particularly for proteoforms with varying modification degrees—remains limited. Our study addresses this gap by assessing PIRR accuracy and precision using well-defined histone H3 proteoforms with site-specfic acetylations. Through orthogonal translation, we produced full-length acetylated proteoforms, precisely quantified them via UV spectrophotometry, and analyzed isometric mixtures by either DI-MS or WCX/HILIC-MS. Our findings show that PIRR quantification aligns with theoretical values across all acetylation degrees and positions, despite potential interference from +2Na adducts or spontaneous acetylations. Given acetylation’s considerable physicochemical effects, these results support PIRR quantification for histone proteoforms with other conventional modifications (e.g., methylations, phosphorylations) at varying degrees. Future implementations should minimize mass interference effects and account for LC binding selectivity effects to enhance the accuracy and sensitivity.

## Supplementary Material











## Notes

Supplementary methods of the recombinant proteoform expression system, protein purification, ETD-MS/MS, and proteoform individual analysis. Supplementary sequence of His6-TEV-H33CM, supplementary figures of pET-11a-His6-TEV-H33CM plasmid, recombinant histone H3 proteoform purification, representative Informed-Proteomics recombinant histone H3 PrSMs, UV spectrophotometry, optimization of AcK incorporation in histone H3 mutants, representative MS scans of targeted proteoform and adduct signals, summary of ETD fragments for recombinant histone H3 proteoforms characterization, DI-MS and WCX/HILIC-MS PIRR quantifications of N-terminal acetylated proteoforms in nonisometric ratios, and DI-MS and WCX/HILIC-MS PIRR quantifications of N-terminal acetylated proteoforms using different charge states for abundance integration (PDF)
Supplementary table of QuikChange mutation primers (XLSX)
Supplementary table of precursor features detected by TopFD in individual proteoform analysess (XLSX)
Supplementary tables of calculated proteoform abundances and PIRRs for isometric mixtures (XLSX)
Supplementary table of calculated proteoform abundances and PIRRs for nonisometric mixtures (XLSX)
