# Supplementary material for: Evaluation of Protein Ion Relative Ratio Quantification in Top-down Electrospray Ionization-Mass Spectrometry Using Site-Specific Acetylated Recombinant Histone H3 Proteoforms
Source: J Am Soc Mass Spectrom. 2025 Sep 5;36(10):2048–58. doi: 10.1021/jasms.5c00079 (PMC12492392; doi:10.1021/jasms.5c00079)
Supplement: Supplementary file 1 [file js5c00079_si_001.pdf]

Supporting Information

Evaluation of Protein Ion Relative Ratio Quantification in Top-down Electrospray Ionization–Mass Spectrometry Using Site-Specific Acetylated Recombinant Histone H3 Proteoforms

Kin-Wing Lui[1], *Sai-Ming, Ngai*[1,2], *Ting-Fung Chan*[1,2,*]

[1] School of Life Sciences, The Chinese University of Hong Kong, Sha Tin, N.T., Hong Kong, 999077

[2] State Key Laboratory of Agrobiotechnology, and School of Life Sciences, The Chinese University of Hong Kong, Shatin N.T., Hong Kong., 999077

*Correspondence: tf.chan@cuhk.edu.hk; Tel.: (+852) 3943-6876

List of Supporting Information

Supplementary Methods

Supplementary Figures

All the supplementary figures can be found in this document.

Sequence S1. DNA and protein sequence of the recombinant histone H3 protein His6-TEV-H33CM
Figure S1. Plasmid map of pET-11a-His6-TEV-H33CM
Figure S2. Purification of the recombinant histone H3
Figure S3. Representative Informed-Proteomics proteoform spectrum matches (PrSMs) of the recombinant histone H3 proteoforms
Figure S4. Absolute quantification of histone H3 proteoforms using UV spectrophotometry
Figure S5. Optimization of N^{ε}-acetyllysine (AcK) incorporation via amber suppression in histone H3 mutants
Figure S6. Enlarged representative MS scans of targeted proteoforms and adduct signals
Figure S7. Summary of ETD fragments for recombinant histone H3 proteoform characterization
Figure S8. DI-MS PIRR quantification of N-terminal acetylated proteoforms in non-isometric ratios
Figure S9. WCX/HILIC-MS PIRR quantification of N-terminal acetylated proteoforms in non-isometric ratios
Figure S10. DI-MS PIRR quantification of N-terminally acetylated proteoforms using different precursor ion charge states for abundance integration
Figure S11. WCX/HILIC-MS PIRR quantification of N-terminal acetylated proteoforms using different precursor ion charge states for abundance integration

Supplementary Tables

All the supplementary tables can be found in the additional spreadsheets.

Table S1. QuikChange Lightning site-directed amber mutation primers
Table S2. Precursor features detected by TopFD in individual proteoform analyses
Table S3. Proteoform abundances and PIRRs for isometric mixtures

Table S4. Proteoform abundances and PIRRs for non-isometric mixtures

Supplementary Methods

Recombinant Proteoform Expression System

$CaCl_2$ Competent Cells Preparation. Targeted cells were grown in LB (with the appropriate antibiotics) at 37 ˚C and 200 rpm until reaching OD_{600} > 0.6. The culture was cooled on ice for 30 mins, followed by centrifugation at 4000× g 5 min. The pellet was resuspended in 100 mM $CaCl_2$ and incubated on ice for 30 min with gentle agitation. After a second centrifugation step, the cells were resuspended in a 15% glycerol/100 mM $CaCl_2$ solution (5% of the original culture volume), aliquoted into 50 μL fractions, snap-frozen in liquid nitrogen, and stored at −80 ˚C.

Plasmid Transformation. Transformation was performed using the heat shock method. Briefly, 40 ng of plasmid was mixed with $CaCl_2$-competent cells and incubated on ice for 15 min. Cells were heat-shocked at 42 ˚C for 30 sec, immediately returned to ice for 2 min, and then recovered in 500 μL SOC medium at 37 ˚C for 1 h (or 1.5 h for cells transformed with the pTECH-chAcK3RS(IPYE) plasmid[1] (AcKRS plasmid)). After recovery, cells were pelleted by centrifugation at 4,000× g for 2 min, resuspended in 50 μL LB, and plated on LB agar containing the appropriate antibiotics. Plates were incubated at 37 ˚C overnight (or for two overnights for AcKRS plasmid transformants).

Protein Expression. For expression of unmodified His6-TEV-H33CM, a single colony of BL21(DE3) cells transformed with pET11a-His6-TEV-H33CM was inoculated into 5 mL LB medium containing 100 μg/mL ampicillin and grown overnight at 37 ˚C with 200 rpm shaking. A 100 μL aliquot of this culture was then transferred to 10 mL fresh LB/Amp medium and incubated under the same conditions until reaching OD600 > 0.6. Protein expression was induced by adding 0.2 mM IPTG, followed by overnight incubation at room temperature with 180 rpm shaking (Fig. S2A "Cell Culture"). Cells were harvested by centrifugation at 4,000× g for 5 min, and the resulting pellets were snap-frozen in liquid nitrogen and stored at −80˚C until further use.

For the expression of acetylated His6-TEV-H33CM, the AcKRS plasmid was first transformed into B-95.ΔA[2] cells. Competent cells prepared from this strain were then transformed with pET-11a-His6-TEV-H33CM plasmid containing the desired amber mutations. A single colony of double-transformed cells was inoculated into 5 mL LB medium supplemented with 100 μg/mL ampicillin and 25 μg/mL chloramphenicol and grown overnight at 37 °C. A 1 mL aliquot of this culture was transferred to 10 mL fresh medium containing 100 μg/mL ampicillin and 3 μg/mL chloramphenicol and grown until OD600 > 0.6. For amber suppression, Nε-acetyl-L-lysine (AcK) was added at concentrations of 20 mM, 100 mM, or 200 mM for constructs containing one, two, or three amber mutations, respectively. AcK (prepared as a 2 M aqueous stock solution and filter-sterilized) was supplemented along with 20 mM nicotinamide (Sigma-Aldrich) to inhibit bacterial deacetylase activity[3]. After 30 minutes of pre-induction incubation at room temperature (180 rpm), protein expression was induced with 0.2 mM IPTG. Subsequent induction and harvesting conditions matched those used for unmodified protein expression.

Protein Purification

Cell lysis and Ni-NTA His-tag purification. Frozen cell pellets from 10 mL cultures were resuspended in 1.2 mL binding buffer (6 M urea, 0.5 M Tris-HCl, 500 mM NaCl, 30 mM imidazole, pH 8.0). The denaturing conditions were necessary to solubilize the recombinant histones, which exhibit poor solubility at neutral pH. Cell lysis was performed on ice using a Sonics Ultrasonic Processor with ten cycles of sonication (5 s pulse, 10 s rest) at 50% amplitude. The lysate was clarified by centrifugation at 12,000× g for 10 min, and the supernatant was collected (Fig. S2A "Lysate"). The supernatant was loaded onto a pre-equilibrated Qiagen Ni-NTA spin column (600 μL binding buffer). After loading, the column was washed once with 600 μL binding buffer, followed by two elutions with 125 μL elution buffer (6 M urea, 0.5 M Tris-HCl, 500 mM NaCl, 300 mM imidazole, pH 8.0) (Fig. S2A "Flow Through", "Wash", and "Elution"). Protein concentration in the eluate was determined

using the Pierce 660 nm Protein Assay. A minimum yield of 150 μg was required for subsequent processing steps.

TEV Digestion. Prior to digestion, the urea concentration was reduced to <1 M by sequential dilution and concentration. The 250 μL eluate was loaded into an Amicon Ultra-0.5 mL 10 kDa MWCO centrifugal filter and mixed with 250 μL TEV buffer (0.5 M Tris-HCl, 500 mM NaCl, pH 8.0). The sample was concentrated to ~400 μL by centrifugation at 12,000× g for 1 min. This was followed by six cycles of 4/5 dilution (adding 100 μL TEV buffer and concentrating to 400 μL), achieving a final urea concentration of ~0.8 M (calculated as 6 × 0.5 × 0.8). After a final centrifugation at 12,000× g for 2 min to remove aggregates, the supernatant was collected for digestion (Fig. S2A "10K MWCO").

For the cleavage reaction, TEV protease (Sigma-Aldrich, ≥2 mg/mL) was added at a ratio of 1 μL per 50 μg target protein. Digestion proceeded at 30 ˚C for 3 h followed by overnight incubation at 4˚C. The reaction mixture was centrifuged at 12,000× g for 2 min to remove any precipitates, yielding supernatant containing predominantly cleaved product with minimal residual full-length protein (Fig. S2A "TEV").

Cleaved Protein Purification. Conventional reverse purification using Ni-NTA resin was avoided due to H33CM's nonspecific binding affinity, which caused significant sample loss. Instead, C4 reversed-phase HPLC (RP-HPLC) was employed. Since RP-HPLC cannot resolve cleaved and uncleaved proteins, complete TEV digestion was essential. Additionally, it was crucial to remove aggregates—primarily containing uncleaved protein that could redissolve in the acidic RP-HPLC buffer—by centrifugation prior to purification.

Before HPLC, the TEV-digested sample was buffer-exchanged into 0.1% formic acid (FA) using a 10 kDa MWCO centrifugal concentrator. The sample was concentrated to ~40 μL by centrifugation at 12,000× g for 15 min, diluted >10-fold with 400 μL 0.1% FA, and

reconcentrated to 40 μL. Finally, the volume was adjusted to ~200 μL with 0.1% FA for HPLC injection.

RP-HPLC separation was performed on a 214TP C4 column (250 mm × 4.6 mm I.D., 5 μm particle size, 300 Å pore size; Grace, now available from VWR) using an Agilent 1260 Infinity system equipped with a standard autosampler (G1329B), quaternary pump (G1311B), thermostatted column compartment (G1316A), diode array detector (G1315D), and fraction collector (G1364C). Buffer A was composed of 0.05% trifluoroacetic acid (TFA) in Milli-Q® water and buffer B was composed of 0.05% TFA in acetonitrile (ACN). The gradient elution program was as follows: 10 – 60% B in 60 mins, 60 – 80% B in 5 mins, isocratic hold at 80% B for 5 mins, 80 – 10% B in 5 mins. Fractions (1 mL/min) were collected from 42–52 min, with the cleaved target eluting at 47 – 49 min (Fig. S2A "HPLC," S2B). Collected fractions were vacuum-dried and stored at −20 °C until use.

ETD-MS/MS Parameters

For proteoform characterization by DI-MS, ETD fragmentation was performed with the following parameters: Top-N mode (3 precursors per duty cycle), monoisotopic peak determination (intact protein mode), charge state filtering (10-30), isolation window 0.4 m/z, Orbitrap resolution 240,000 (FWHM at 200 m/z), scan range 200-2000 m/z, normalized AGC target 2000% (equivalent to 1e6 ions), maximum injection time 500 ms, 3 microscans, and profile mode data acquisition. ETD conditions included a 5 ms reaction time with a reagent target of 1e6 ions and maximum injection time of 200 ms.

For WCX/HILIC-MS analyses, similar MS/MS parameters were employed with the following optimizations to accommodate chromatographic elution: Orbitrap resolution reduced to 120,000, maximum injection time set to 246 ms, and 1 microscan. To address co-eluting species, Top-N mode was increased to 5 precursors per duty cycle.

Proteoform Individual Analysis

The proteoform individual analysis were searched using Informed Proteomics[4] (version 1.0.7017, https://github.com/PNNL-Comp-Mass-Spec/Informed-Proteomics). Raw spectra data were converted to .mzML format using MSConvert[5] (version 3.0.24117-481ff6c, https://proteowizard.sourceforge.io/) prior to analysis with Informed-Proteomics. The search parameters included: (1) a custom FASTA sequence of H33CM (lacking the His6-TEV tag, beginning with glycine at position 1); (2) variable lysine acetylation modifications (maximum 5 modifications per proteoform); (3) mass range of 15,000-16,000 Da; (4) precursor charge states 2-50; and (5) 10 ppm mass tolerance. Internal cleavage, tag search, and decoy database options were disabled during the search.

For purity assessment, individual proteoform samples were analyzed using TopFD[6] (version 1.7.8, https://www.toppic.org/software/toppic/index.html) with the following deconvolution parameters: 0.02 m/z peak error tolerance, S/N threshold of 3 for both MS1 and MS/MS spectra, 0.4 m/z precursor isolation window, maximum charge state of 30, minimum of 3 scans per feature, disabled MS-Deconv scoring, and an ECScore cutoff of 0.9 with final filtering enabled. Processed features (_ms1.feature files) were further filtered by ECScore ≥ 0.99 before relative abundance calculations (Table S2).

Supplementary Figures

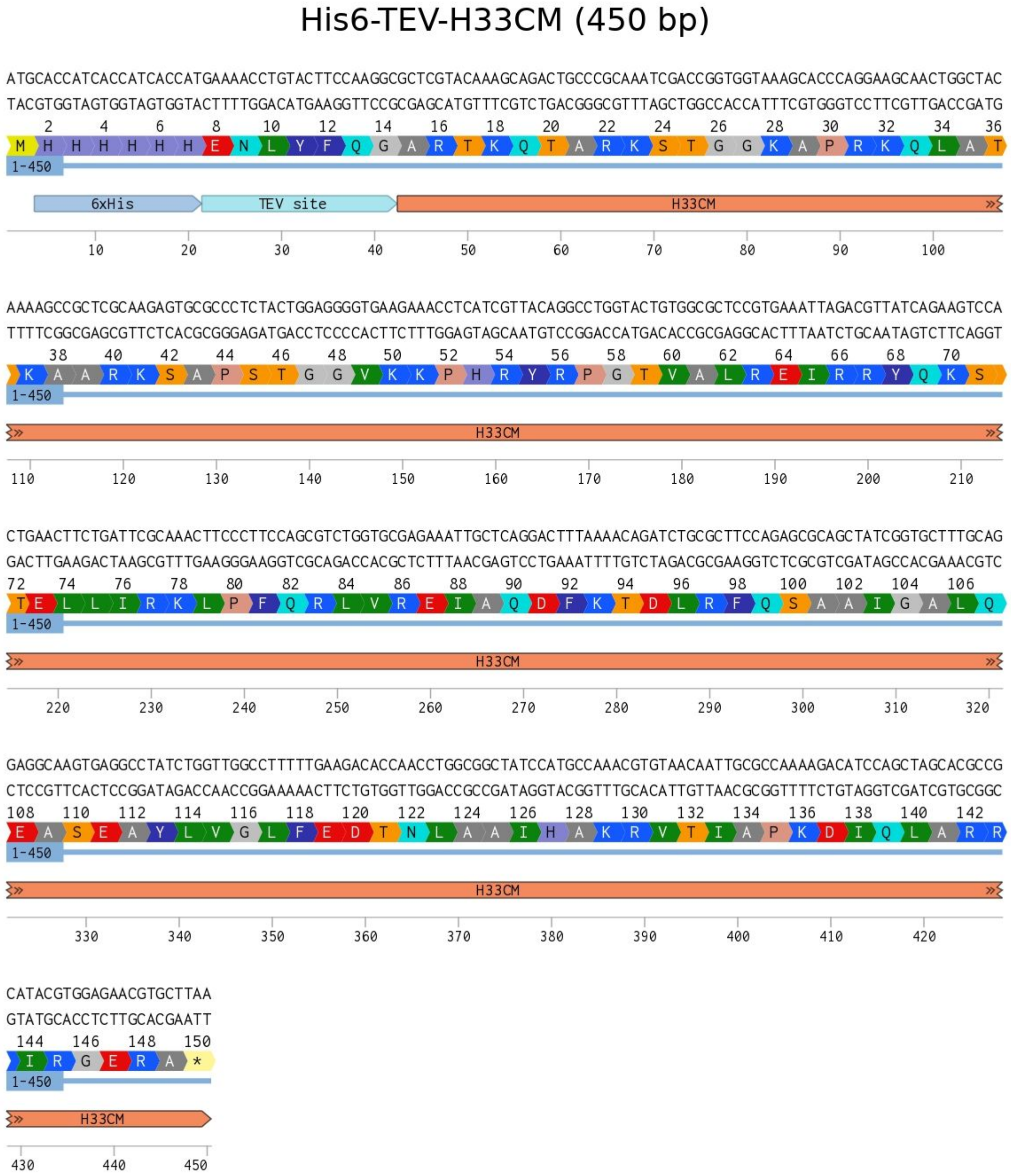

Sequence S1. DNA and protein sequence of the recombinant histone H3 protein His6-TEV-H33CM. The TEV protease cleavage site (ENLYFQ↓G) generates purified H33CM with an N-terminal glycine. While this study uses canonical histone H3 numbering, all residue positions in H33CM are offset by +1 relative to the canonical sequence.

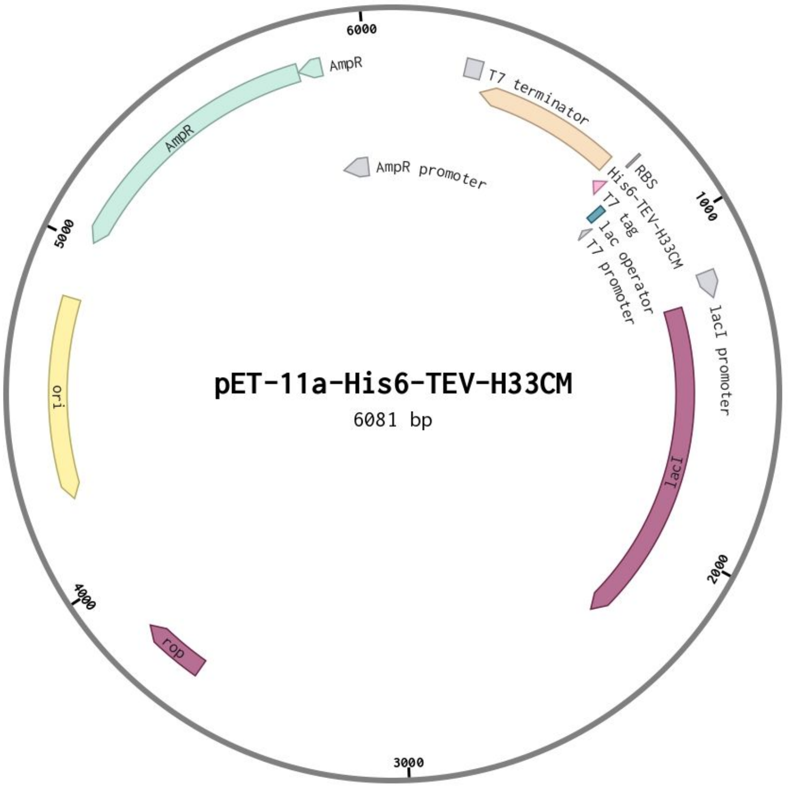

Figure S1. Plasmid map of pET-11a-His6-TEV-H33CM.

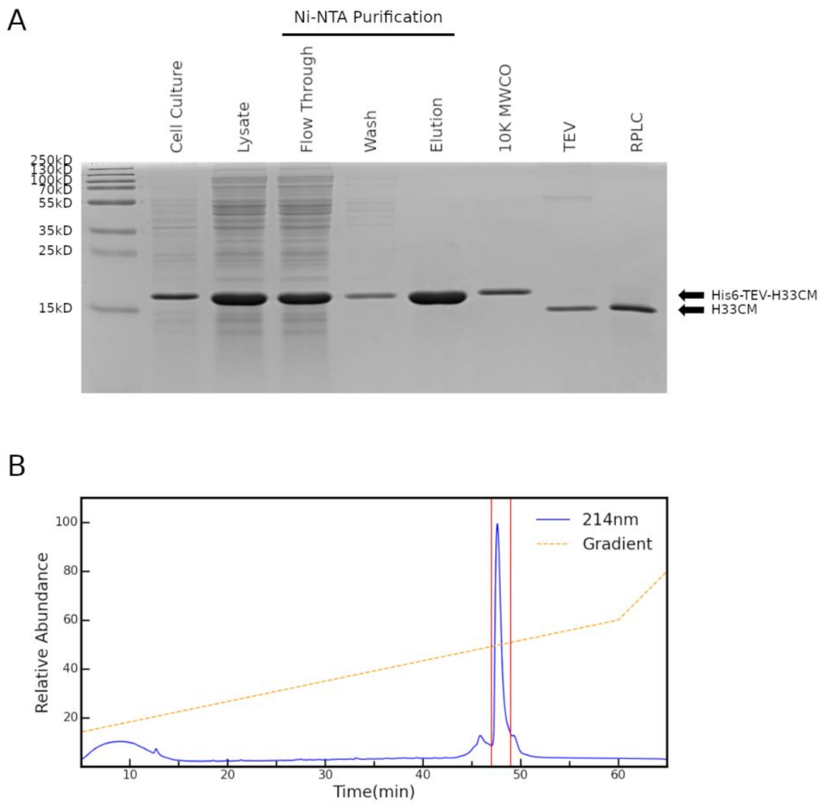

Figure S2. Purification of the recombinant histone H3. (A) SDS-PAGE (Coomassie Blue G250 stain) analysis of key purification steps: *Cell culture* (IPTG-induced overnight culture); *Lysate* (sonicated supernatant); *Flow-through*, *Wash*, and *Elution* (Ni-NTA purification fractions); *10K MWCO* (urea-reduced sample); *TEV* (digested product after 3 h at 30˚C + overnight at 4˚C); *RPLC* (final purified H33CM). All lanes loaded with 5 μL sample from unmodified H33CM purification. (B) RP-HPLC chromatogram (214 nm absorbance, blue trace) of TEV-digested sample with acetonitrile gradient (orange dashed line, % B). The red box highlights the collected fraction containing target H33CM (unmodified proteoform shown as representative example).

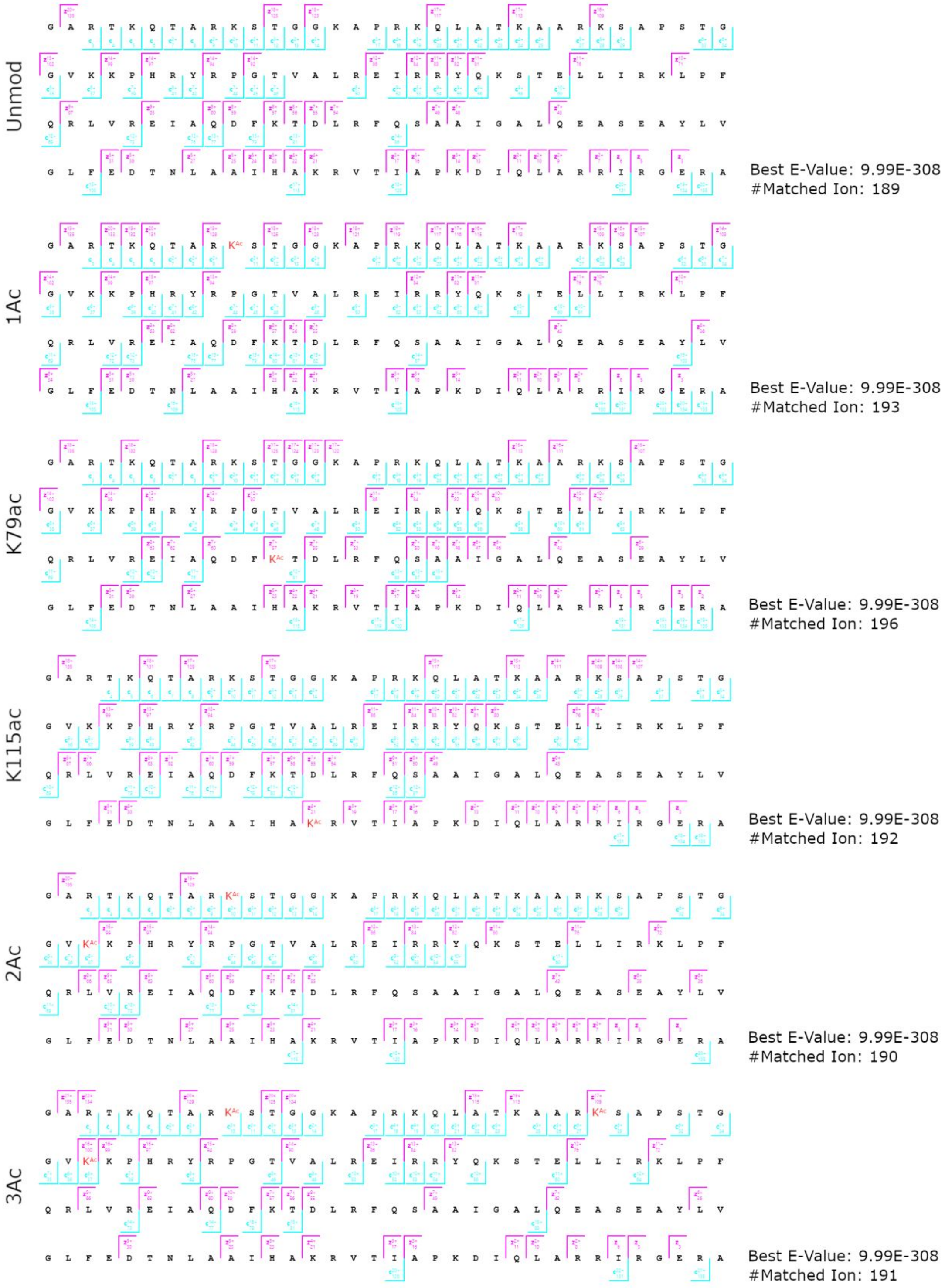

Figure S3. Representative Informed-Proteomics proteoform spectrum matches (PrSMs) of the recombinant histone H3 proteoforms. Acetylation sites are manually annotated for clarity. For each proteoform, the best-match PrSM is shown with its corresponding E-value and number of matched fragment ions (right). The N-terminal glycine originates from TEV protease cleavage of the His6-TEV tag.

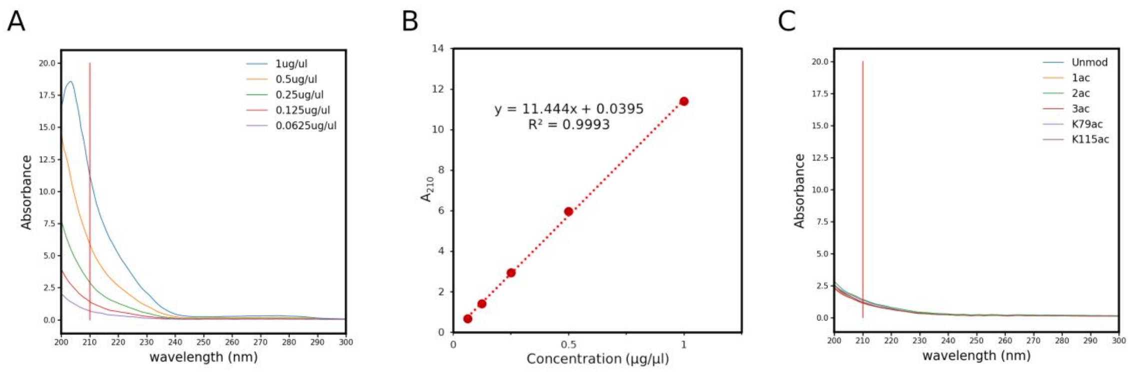

Figure S4. Absolute quantification of histone H3 proteoforms using UV spectrophotometry. (A) UV spectra (200-300 nm) of commercial histone H3.1 standards at varying concentrations. The red line indicates the 210 nm wavelength selected for quantification. (B) Calibration curve derived from 210 nm absorbance values in (A). (C) Comparative UV spectra of acetylated H33CM proteoforms after normalization to 0.1 μg/μL. Identical y-axis scaling in (A) and (C) validates sample measurements against the standard reference.

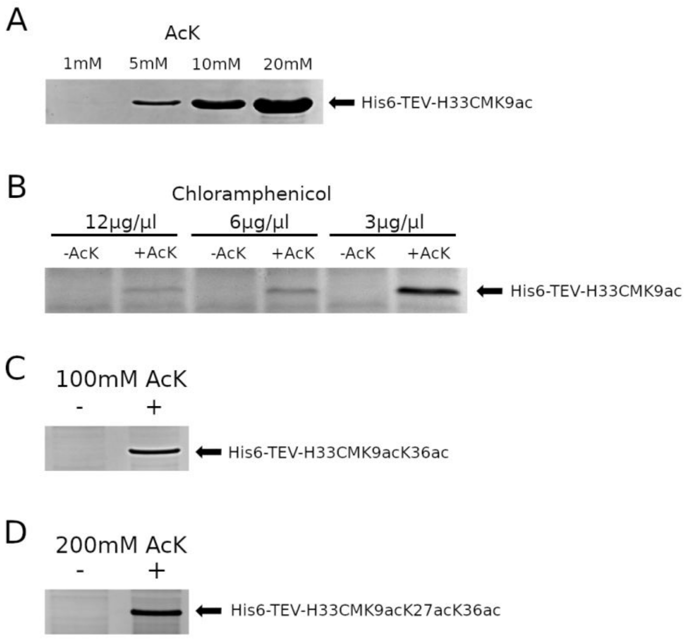

Figure S5. Optimization of N^{ε}-acetyllysine (AcK) incorporation via amber suppression in histone H3 mutants. (A) AcK concentration screen (0-20 mM) for His6-TEV-H33CMK9ac expression (5 µL Ni-NTA elution analyzed by SDS-PAGE). (B) Chloramphenicol concentration test (3-25 µg/mL) with/without 10 mM AcK for His6-TEV-H33CMK9ac. (C–D) Expression analysis of doubly (K9acK36ac; C) and triply (K9acK27acK36ac; D) mutated constructs with/without 100 mM or 200 mM AcK, respectively. For B–D, 5 µL of whole-cell lysate from overnight-induced cultures was analyzed by SDS-PAGE.

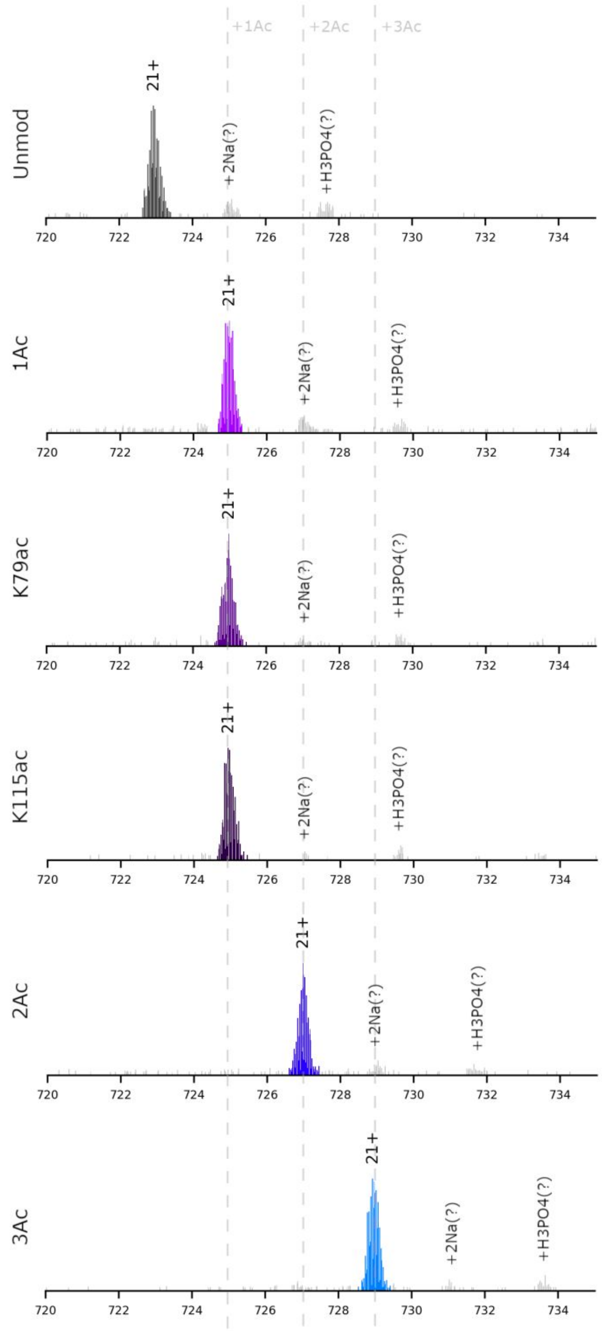

Figure S6. Enlarged representative MS scans of targeted proteoforms and adduct signals. Detailed views display the *m/z* range containing 21+ precursor ions, annotated to show: (1) targeted proteoforms (color-matched to Fig. 1); (2) acetylation mass shifts (+42.01 Da, light gray dashed lines); and (3) off-target adducts ("+2Na(?)" for sodium adducts (+43.96 Da), "+H3PO4(?)" for phosphoric acid adducts (+97.98 Da)). In mixed samples, +2Na of any n-acetylated proteoform interfere with the (n+1)-acetylated proteoform due to their close mass proximity.

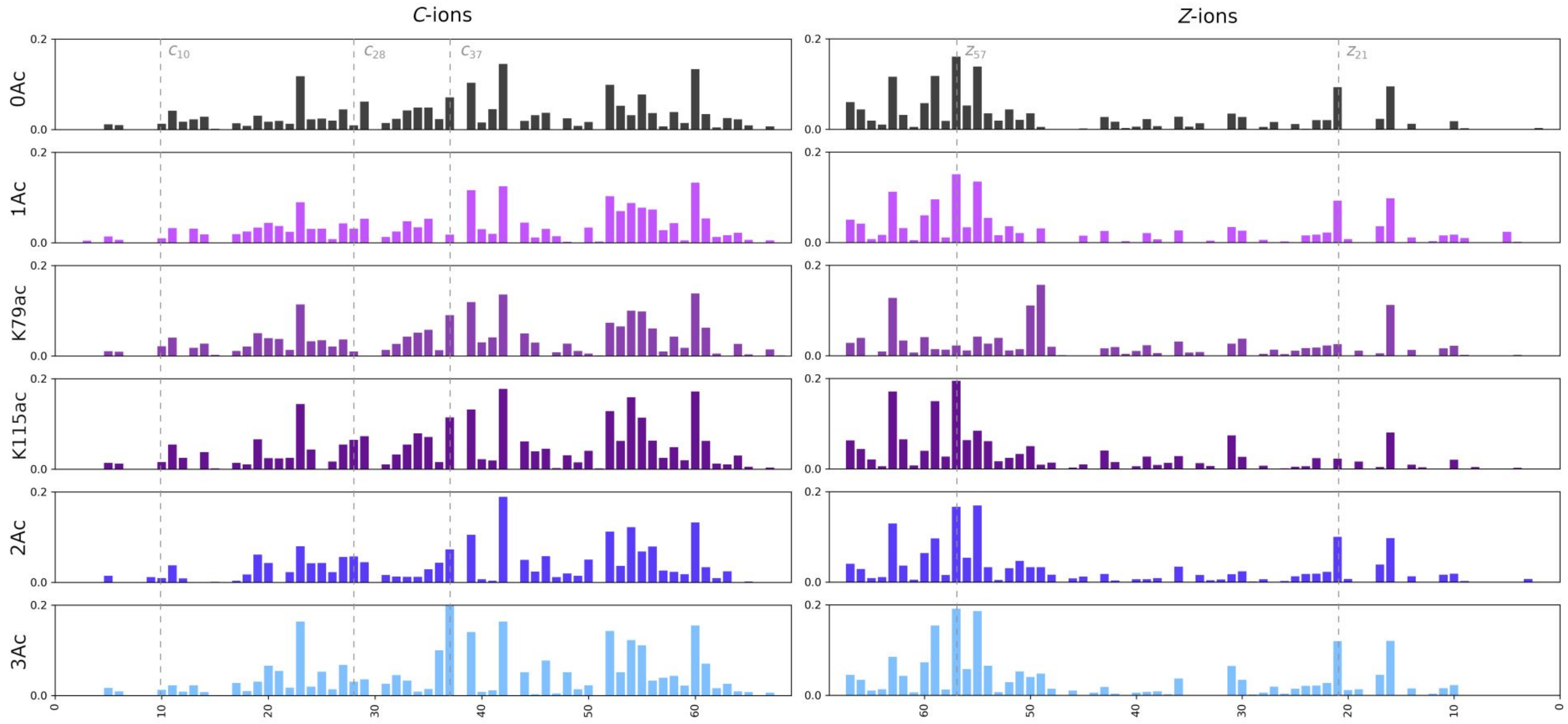

Figure S7. Summary of ETD fragments for recombinant histone H3 proteoform characterization. Averaged MS/MS spectra (FreeStyle software) were deconvoluted to monoisotopic masses (TopFD) and matched to *c*/*z*-type fragment ions (20 ppm mass tolerance). Five characteristic ions (c_{10}, c_{28}, c_{37}, z_{57}, z_{21}) that could potentially localize acetylation sites are indicated. While these fragments would theoretically serve as ideal references, c_{28} and c_{37} ions had inconsistent charge state distributions across proteoforms, preventing direct *m/z* comparisons in raw spectra. Therefore, Fig. 1 instead displays alternative ions (c_{31}^{5+}, c_{39}^{6+}) that maintained consistent charge states for cross-proteoform comparison.

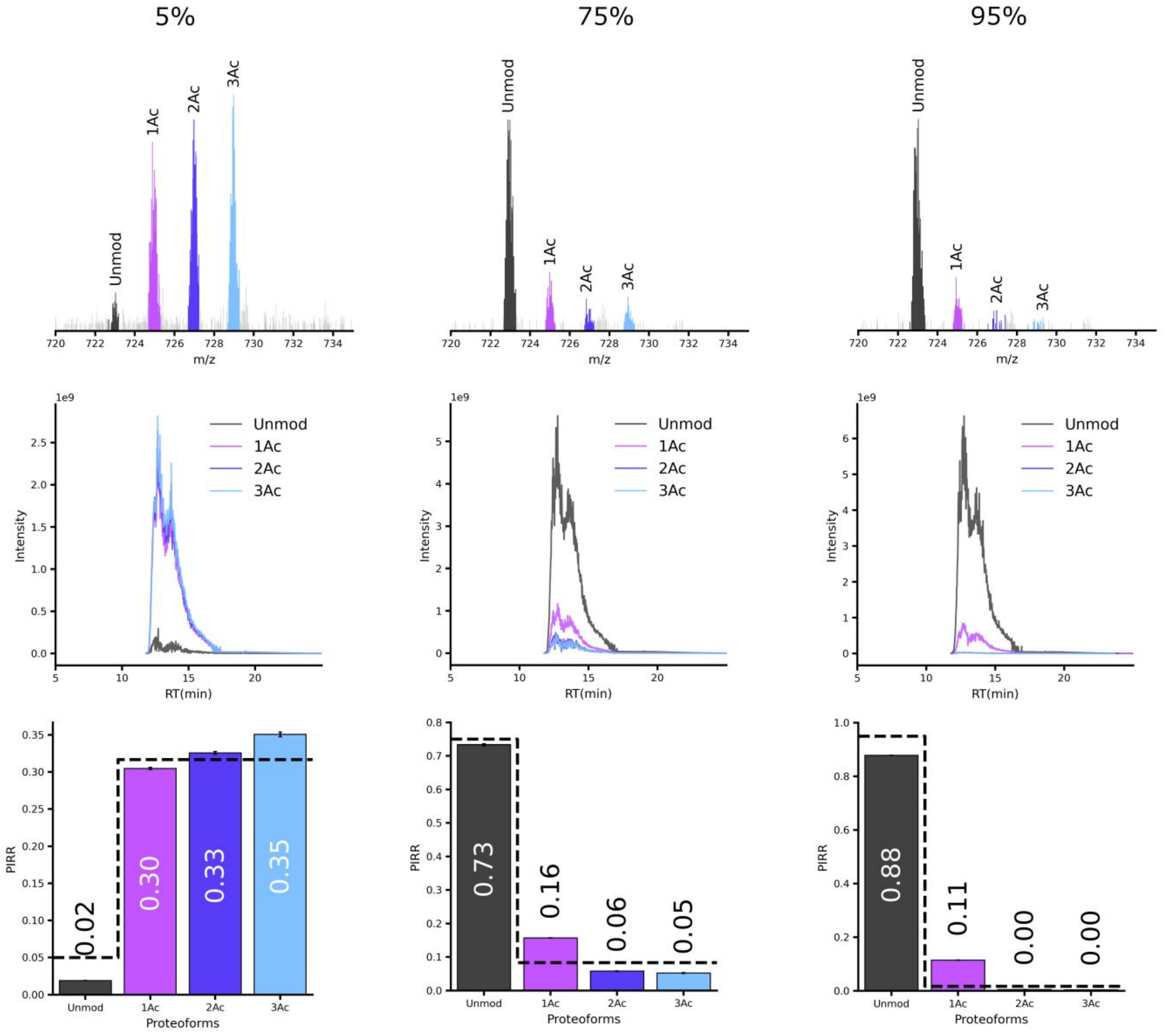

Figure S8. DI-MS PIRR quantification of N-terminal acetylated proteoforms in non-isometric ratios. Using the same visualization format as Fig. 2, samples containing 5%, 75%, or 95% unmodified histone H3 were analyzed to evaluate quantification accuracy across dynamic ranges. Signal interference affecting 1Ac proteoforms intensified with higher unmodified proteoform abundance. Theoretical PIRRs distributions were 5% unmod [0.05, 0.316, 0.316, 0.316]; 75% unmod [0.75, 0.083, 0.083, 0.083]; 95% unmod [0.95, 0.0166, 0.0166, 0.0166] (black dashed lines in plots). Complete quantification metrics (abundances, PIRRs, absolute/percent errors, CVs) are provided in Table S3.

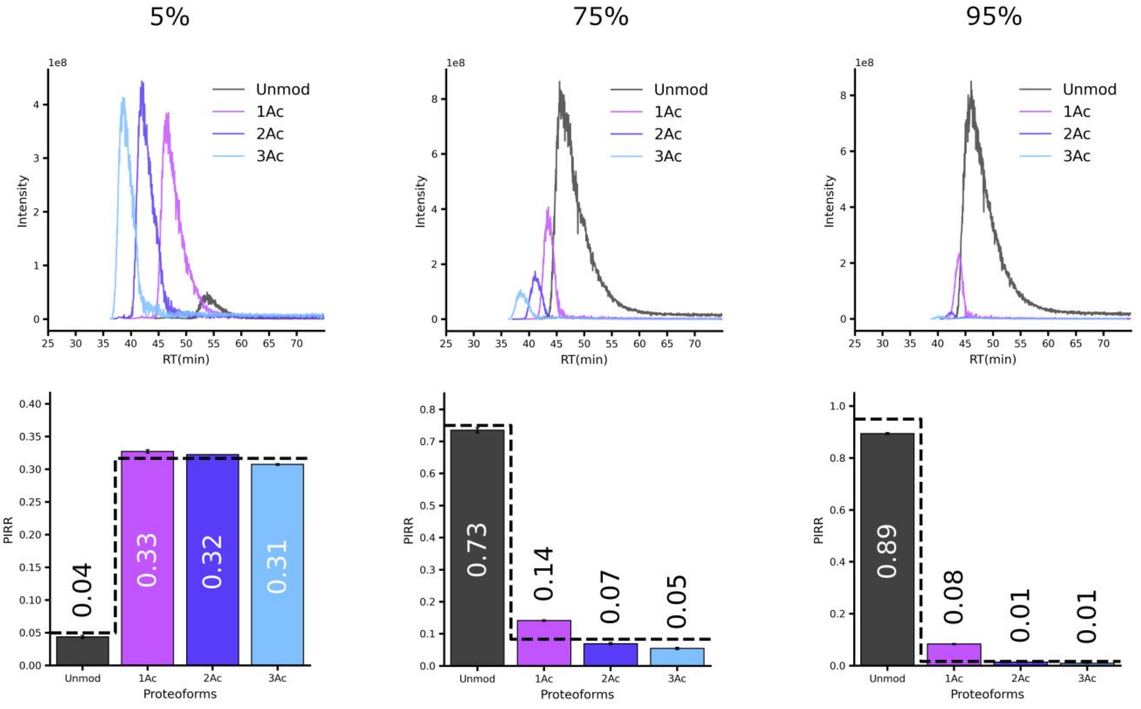

Figure S9. WCX/HILIC-MS PIRR quantification of N-terminal acetylated proteoforms in non-isometric ratios. Analysis followed the same format as Fig. 3, revealing unexpected 1Ac proteoform signal interference. The enhanced 1Ac LC peaks suggest either: (1) +2Na adducts exhibiting retention behavior similar to acetylated forms, or (2) host-derived spontaneous acetylation. ETD-MS/MS analysis (Informed-Proteomics) confirmed the interfering signals lacked acetylation at expected sites, ruling out cross-contamination. Theoretical PIRR values (identical to Fig. S8) are shown as black dashed lines. Complete quantification data (abundances, PIRRs, errors, CVs) are provided in Table S4.

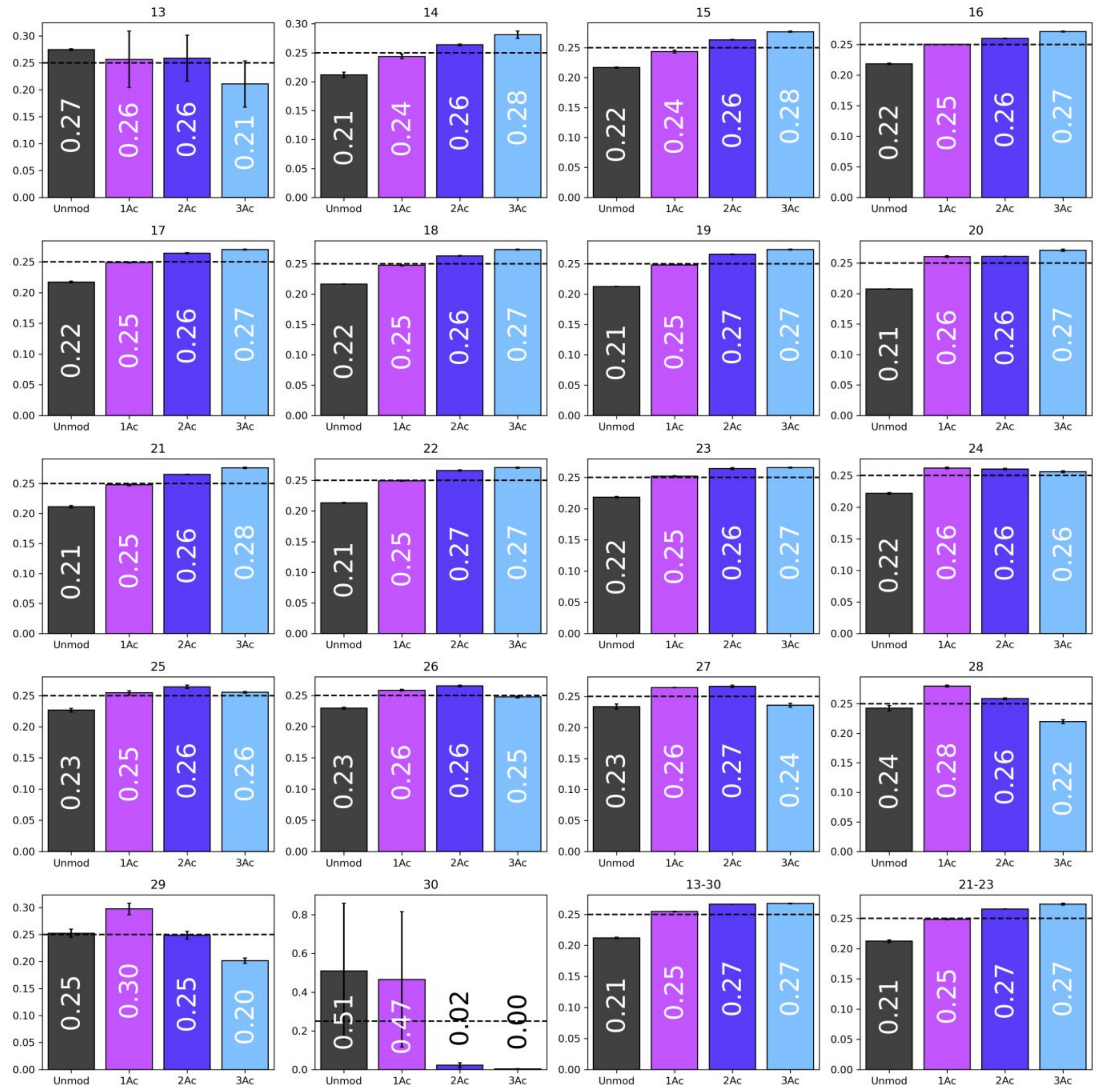

Figure S10. DI-MS PIRR quantification of N-terminally acetylated proteoforms using different precursor ion charge states for abundance integration. Bar plots compare PIRR values obtained from: individual charge states (13+ to 30+), the three most intense charge states (21+ to 23+), and all charge states combined. Most charge states (excluding extreme 13+ and 30+ cases) and both integrated approaches (all charges or top three) yield consistent results. Each graph plots experimental PIRRs (y-axes) with theoretical ratios indicated as black dashed lines. Graph titles indicate which charge states were included in each analysis.

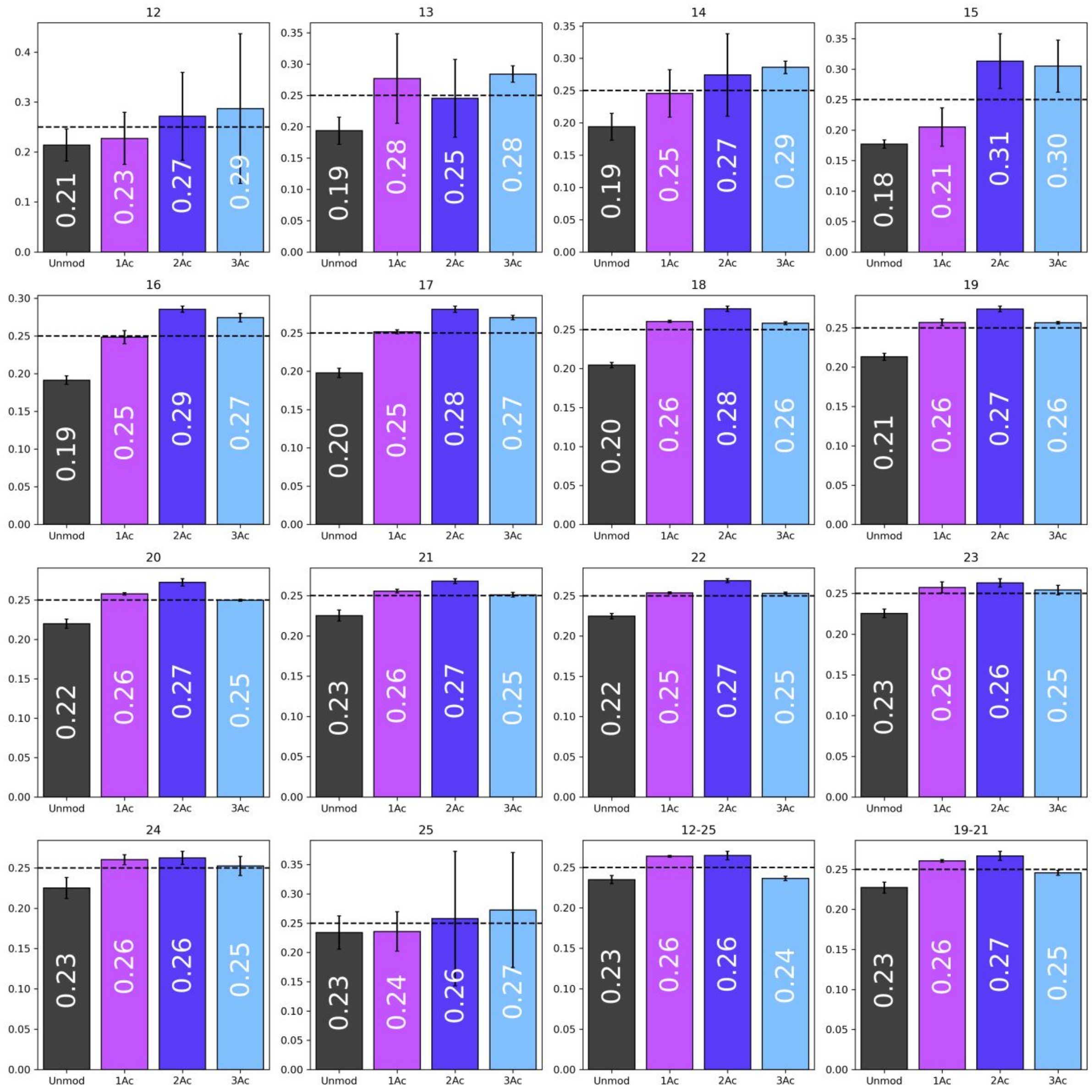

Figure S11. WCX/HILIC-MS PIRR quantification of N-terminal acetylated proteoforms using different precursor ion charge states for abundance integration. Compared to DI-MS (Fig. S10), charge state distributions shifted to 12+ to 25+, with 19+ to 21+ being most intense. PIRR values were calculated from: individual charge states, the top three charge states (19+ to 21+), and all charge states combined. Each graph plots experimental PIRRs (y-axes) with theoretical ratios indicated as black dashed lines. Graph titles indicate which charge states were included in each analysis.

REFERENCES

(1) Bryson, D. I.; Fan, C.; Guo, L.-T.; Miller, C.; Söll, D.; Liu, D. R. Continuous Directed Evolution of Aminoacyl-tRNA Synthetases. *Nature Chemical Biology* **2017**, *13* (12), 1253–1260. https://doi.org/10.1038/nchembio.2474.

(2) Mukai, T.; Hoshi, H.; Ohtake, K.; Takahashi, M.; Yamaguchi, A.; Hayashi, A.; Yokoyama, S.; Sakamoto, K. Highly Reproductive Escherichia Coli Cells with No Specific Assignment to the UAG Codon. *Sci Rep* **2015**, *5*, 9699. https://doi.org/10.1038/srep09699.

(3) Neumann, H.; Peak-Chew, S. Y.; Chin, J. W. Genetically Encoding Nε -Acetyllysine in Recombinant Proteins. *Nature Chemical Biology* **2008**, *4* (4), 232–234. https://doi.org/10.1038/nchembio.73.

(4) Park, J.; Piehowski, P. D.; Wilkins, C.; Zhou, M.; Mendoza, J.; Fujimoto, G. M.; Gibbons, B. C.; Shaw, J. B.; Shen, Y.; Shukla, A. K.; Moore, R. J.; Liu, T.; Petyuk, V. A.; Tolić, N.; Paša-Tolić, L.; Smith, R. D.; Payne, S. H.; Kim, S. Informed-Proteomics: Open-Source Software Package for Top-down Proteomics. *Nat Methods* **2017**, *14* (9), 909–914. https://doi.org/10.1038/nmeth.4388.

(5) Chambers, M. C.; Maclean, B.; Burke, R.; Amodei, D.; Ruderman, D. L.; Neumann, S.; Gatto, L.; Fischer, B.; Pratt, B.; Egertson, J.; Hoff, K.; Kessner, D.; Tasman, N.; Shulman, N.; Frewen, B.; Baker, T. A.; Brusniak, M.-Y.; Paulse, C.; Creasy, D.; Flashner, L.; Kani, K.; Moulding, C.; Seymour, S. L.; Nuwaysir, L. M.; Lefebvre, B.; Kuhlmann, F.; Roark, J.; Rainer, P.; Detlev, S.; Hemenway, T.; Huhmer, A.; Langridge, J.; Connolly, B.; Chadick, T.; Holly, K.; Eckels, J.; Deutsch, E. W.; Moritz, R. L.; Katz, J. E.; Agus, D. B.; MacCoss, M.; Tabb, D. L.; Mallick, P. A Cross-Platform Toolkit for Mass Spectrometry and Proteomics. *Nat Biotechnol* **2012**, *30* (10), 918–920. https://doi.org/10.1038/nbt.2377.

(6) Kou, Q.; Xun, L.; Liu, X. TopPIC: A Software Tool for Top-down Mass Spectrometry-Based Proteoform Identification and Characterization. *Bioinformatics* **2016**, *32* (22), 3495–3497. https://doi.org/10.1093/bioinformatics/btw398.
